# Genetic Associations With White Matter Hyperintensities Confer Risk of Lacunar Stroke

**DOI:** 10.1161/STROKEAHA.115.011625

**Published:** 2016-04-25

**Authors:** Matthew Traylor, Loes C.A. Rutten-Jacobs, Vincent Thijs, Elizabeth G. Holliday, Chris Levi, Steve Bevan, Rainer Malik, Giorgio Boncoraglio, Cathie Sudlow, Peter M. Rothwell, Martin Dichgans, Hugh S. Markus

**Affiliations:** From the Department of Clinical Neurosciences, University of Cambridge, Cambridge, United Kingdom (M.T., L.C.A.R.-J., H.S.M.); Department of Medical and Molecular Genetics, King’s College London, London, United Kingdom (M.T.); Laboratory of Neurobiology, Vesalius Research Center, VIB, Experimental Neurology and Leuven Research Institute for Neuroscience and Disease, University of Leuven, Leuven, Belgium (V.T.); Department of Neurology, Austin Health and Florey Institute of Neuroscience and Mental Health, Heidelberg, VIC, Australia (V.T.); School of Medicine and Public Health (E.G.H.) and Centre for Clinical Epidemiology and Biostatistics, Hunter Medical Research Institute and School of Medicine and Public Health (C.L.), University of Newcastle, Newcastle, NSW, Australia; Clinical Research Design, IT and Statistical Support Unit, Hunter Medical Research Institute, New Lambton Heights, NSW, Australia (E.G.H.); School of Life Science, University of Lincoln, Lincoln, United Kingdom (S.B.); Institute for Stroke and Dementia Research, Klinikum der Universität München, Ludwig-Maximilians-University, Munich, Germany (R.M., M.D.); Department of Cerebrovascular Disease, IRCCS Istituto Neurologico Carlo Besta, Milan, Italy (G.B.); Division of Clinical Neurosciences, Neuroimaging Sciences and Institute of Genetics and Molecular Medicine, University of Edinburgh, Edinburgh, United Kingdom (C.S.); Stroke Prevention Research Unit, Nuffield Department of Neuroscience, University of Oxford, Oxford, United Kingdom (P.M.R.); and Munich Cluster for Systems Neurology (SyNergy), Munich, Germany (M.D.).

**Keywords:** cerebral small vessel diseases, genetics, genetic association studies, leukoencephalopathies, stroke, lacunar

## Abstract

**Background and Purpose—:**

White matter hyperintensities (WMH) are increased in patients with lacunar stroke. Whether this is because of shared pathogenesis remains unknown. Using genetic data, we evaluated whether WMH-associated genetic susceptibility factors confer risk of lacunar stroke, and therefore whether they share pathogenesis.

**Methods—:**

We used a genetic risk score approach to test whether single nucleotide polymorphisms associated with WMH in community populations were associated with magnetic resonance imaging–confirmed lacunar stroke (n=1,373), as well as cardioembolic (n=1,331) and large vessel (n=1,472) Trial of Org 10172 in Acute Stroke Treatment subtypes, against 9,053 controls. Second, we separated lacunar strokes into those with WMH (n=568) and those without (n=787) and tested for association with the risk score in these 2 groups. In addition, we evaluated whether WMH-associated single nucleotide polymorphisms are associated with lacunar stroke, or in the 2 groups.

**Results—:**

The WMH genetic risk score was associated with lacunar stroke (odds ratio [OR; 95% confidence interval [CI]]=1.14 [1.06–1.22]; *P*=0.0003), in patients both with and without WMH (WMH: OR [95% CI]=1.15 [1.05–1.26]; *P*=0.003 and no WMH: OR [95% CI]=1.11 [1.02–1.21]; *P*=0.019). Conversely, the risk score was not associated with cardioembolic stroke (OR [95% CI]=1.03 [0.97–1.09]; *P*=0.63) or large vessel stroke (OR [95% CI]=0.99 [0.93,1.04]; *P*=0.39). However, none of the WMH-associated single nucleotide polymorphisms passed Bonferroni-corrected significance for association with lacunar stroke.

**Conclusions—:**

Genetic variants that influence WMH are associated with an increased risk of lacunar stroke but not cardioembolic or large vessel stroke. Some genetic susceptibility factors seem to be shared across different radiological manifestations of small vessel disease.

## Abstract 2

Supplemental Digital Content is available in the text.

Cerebral small vessel disease (SVD) affects the small perforating arteries of the brain and is characterized radiologically by several features, including white matter hyperintensities (WMH), subcortical lacunar infarcts, intracerebral hemorrhages, and cerebral microbleeds.^1^ Despite the considerable impact of SVD on health through increased risk of stroke and vascular dementia, the pathophysiological mechanisms underlying SVD remain largely unknown. Pathological findings in diseased vessels include lipohyalinosis and microatheroma,^2,3^ whereas in the parenchyma findings include myelin pallor, enlargement of perivascular spaces, and gliosis.^4^ Many of these findings are common to both WMH and lacunar stroke.^5^ However, pathological studies have been hampered by methodological and phenotypic inconsistencies.^5^ In addition, little is known about the extent to which underlying pathogenesis is shared across the radiological manifestations. WMH are increased in lacunar stroke^6,7^; more so than in other pathological subtypes of stroke, which may indicate that shared pathological processes underlie the 2. In addition, both confluent WMH and lacunar infarcts are a common finding in Mendelian forms of SVD such as cerebral autosomal dominant arteriopathy with subcortical infarcts and leukoencephalopathy,^8^ although the underlying arterial pathology is considerably different to that of sporadic SVD. However, aside from these exceptions and shared cardiovascular risk factors such as hypertension, few molecular processes have been robustly shown to impact on both lacunar stroke and WMH.

Genetic studies can provide novel insights into SVD and the nature of the relationship between its manifestations. In particular, genome-wide association studies have recently identified multiple genetic variants associated with WMH in community-dwelling individuals^9^ and have been used to show that common variants in *COL4A2*, a gene associated with monogenic SVD, influence sporadic SVD.^10^ In addition, genome-wide association studies provide a means of interrogating the relationship between complex traits and assessing whether such traits share pathogenesis. Polygenic risk score approaches can be used to investigate whether 2 conditions are genetically related by testing whether the cumulative effect of trait-associated single nucleotide polymorphisms (SNPs) associated with the first trait influence a second trait. Such approaches have previously been used to assess the influence of risk factors on stroke^11,12^ and to assess the shared susceptibility between stroke and migraine.^13^

In this analysis, we evaluated the impact of common genetic variants associated with WMH from community populations on the risk of lacunar stroke in a well-characterized population of magnetic resonance imaging (MRI)–confirmed lacunar stroke cases and controls. As heterogeneity in the pathology underlying lacunar stroke has been hypothesized,^14^ and to test whether an association with lacunar stroke was present in individuals without substantial WMH, we separated our lacunar stroke cases into those with substantial WMH and those with no or mild WMH, testing the influence of WMH-associated variants on these subgroups, as well as on cardioembolic and large vessel strokes. We first used a genetic risk score approach to evaluate the overall evidence that WMH-associated variants affect stroke phenotypes, and then second evaluated whether each of the specific genetic variants is associated with lacunar stroke in both the groups with and without WMH.

## Materials and Methods

### Study Participants

The study data set consisted of stroke cases obtained from hospital admissions in the UK and Germany (DNA-lacunar, Genes and Ischaemic Stroke [GENESIS] and Wellcome Trust Case Control Consortium 2 [WTCCC2] study), Australia (Australian Stroke Genetics Collaborative [ASGC]), Italy (Milano—Besta Stroke Register [BSR]), and Belgium (Leuven Stroke Study [LSS]), as well as 9053 controls consisting of ancestry-matched individuals from each of the respective case populations (Table). These data sets have been described in detail in previous publications.^15–18^ Genotyping and imputation of the individuals are described in the online-only Data Supplement. Briefly, all data sets were genotyped on commercially available Illumina arrays and imputed to 1000 Genomes phase 3 using SHAPEIT v2 (for phasing) and IMPUTE v2.2.2 (for imputation).

**Table.**
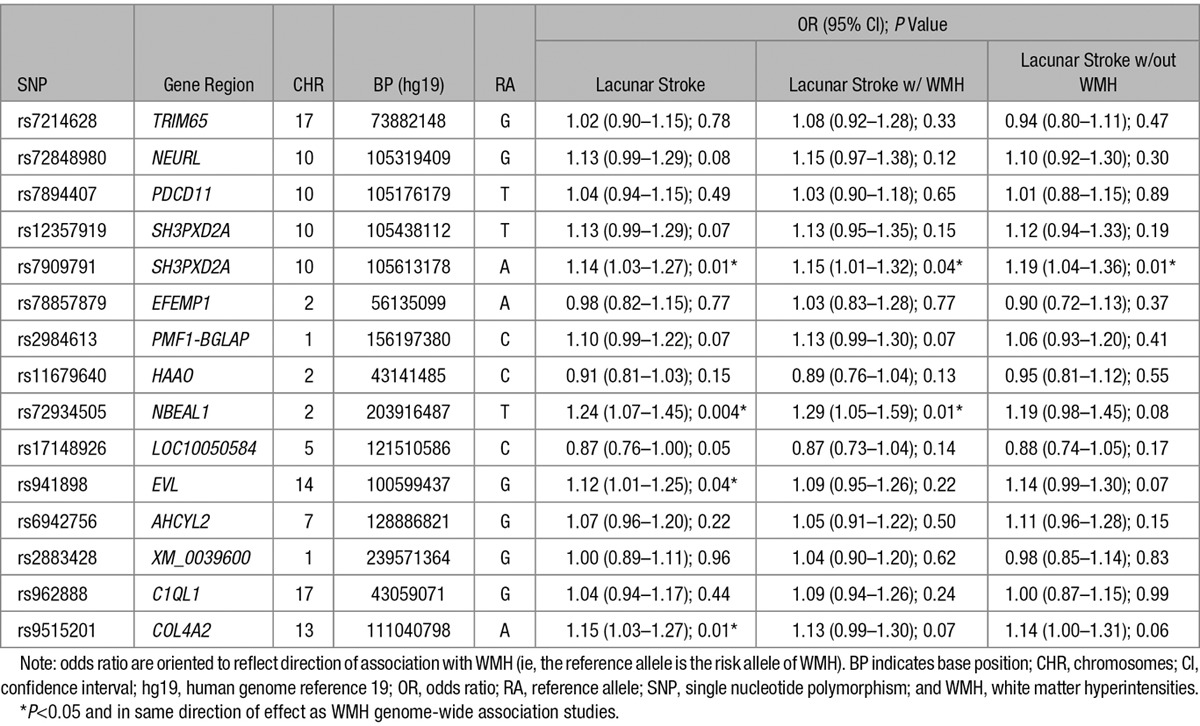
Association of SNPs Associated With WMH in Lacunar Stroke Phenotypes

### Phenotype Classification

Subtyping of the ASGC, WTCCC2, GENESIS, BSR, and LSS groups was initially performed using the Trial of Org 10172 in Acute Stroke Treatment (TOAST) classification,^19^ using clinical assessment as well as brain and vascular imaging where available. For this analysis, we considered only cardioembolic, large vessel, and small vessel subtypes. For the cases that were classified as small vessel stroke under TOAST and had accompanying MRI, as well as all cases from UK Young Lacunar Stroke Study (DNA-lacunar) which included only cases with the TOAST SVD subtype, we performed further characterization, as follows. All MRI scans were centrally reviewed by 1 physician (H.S.M.). The diagnosis of lacunar stroke was confirmed using the following criteria. Lacunar stroke was defined as a clinical lacunar syndrome,^20^ with an anatomically compatible lesion on MRI (subcortical infarct ≤15 mm in diameter). For MRIs performed in the acute phase, the diagnosis was made by an acute lacunar infarct on DWI. For scans not performed in the acute phase, the diagnosis was made by a lacunar syndrome in combination with a lacunar infarct visualized on T1 and fluid-attenuated inversion recovery as a cavitated lesion in an anatomically appropriate location. Exclusion criteria were as follows: stenosis >50% in the extra- or intracranial cerebral vessels; cardioembolic source of stroke, defined according to the TOAST criteria as high or moderate probability; subcortical infarct >15 mm in diameter, as these can be caused by embolic mechanisms (striatocapsular infarcts); any other specific cause of stroke (eg, lupus anticoagulant, cerebral vasculitis, dissection, monogenic forms of stroke, such as cerebral autosomal dominant arteriopathy with subcortical infarcts and leukoencephalopathy). For each individual with a lacunar stroke, we characterized the degree of WMH using the semiquantitative Fazekas scale,^21^ which classifies individuals into 4 groups ranging from none (0) to severe (3). Based on this grading, we then divided the lacunar stroke cases into those with and without WMH: (1) no WMH—patients with only mild or absent leukoaraiosis (Fazekas grade 0 or 1) and (2) WMH—patients with moderate or severe leukoaraiosis (Fazekas grade ≥2).

### Genetic Risk Score Analyses

For each of the 18 SNPs associated with WMH in community populations in a recent study (8 genome-wide and 10 with *P*<1×10^−5^ in Europeans or overall),^9^ we generated an unweighted risk score for each individual in our data set by counting the number of risk alleles and summing across all SNPs. We used an unweighted approach, rather than an approach weighted on the log of the odds ratio (OR), as effect sizes were not reported for the published associations with WMH.^9^ Three of the SNPs (rs186314186, rs150695384, and rs117126031) were rare and not well imputed in our data set so were not included. Within each data set, we then converted each individual’s risk score to a *Z*-score using the standard transformation. We then used logistic regression to estimate the influence of the risk score on each stroke outcome, including ancestry-informative principal components to control for population stratification and meta-analyzing the results using a fixed-effects inverse variance weighted approach. We tested for association of the genetic risk score with lacunar, cardioembolic, and large vessel stroke.

To investigate whether an association with lacunar stroke was independent of WMH, we performed the same analysis on 2 subgroups of lacunar stroke stratified on the presence of substantial WMH (Fazekas ≥2). As the purpose of this analysis was to identify whether the observed association with lacunar stroke was independent of WMH, we did not perform the same analyses in the other subtypes. All ORs reported are per 1 SD change in the normally distributed risk score. We set the criteria for statistical significance at *P*<0.01, Bonferroni-correcting for the 5 tests.

### Single SNP Analyses

In addition, we tested the association of each of the 15 available SNPs with lacunar stroke and the 2 subgroups based on the presence or absence of WMH. For each SNP we performed analyses separately in the 3 batches, including the first 10 ancestry-informative principal components in each analysis. We meta-analyzed the results using a fixed-effects inverse-variance weighted approach. We set the significance threshold at *P*<0.0011, correcting for the 15 SNPs in each of the 3 phenotypes (45 tests in total). All analyses were performed using the R statistical software.

## Results

### Cohort Characteristics

**Figure 1.**
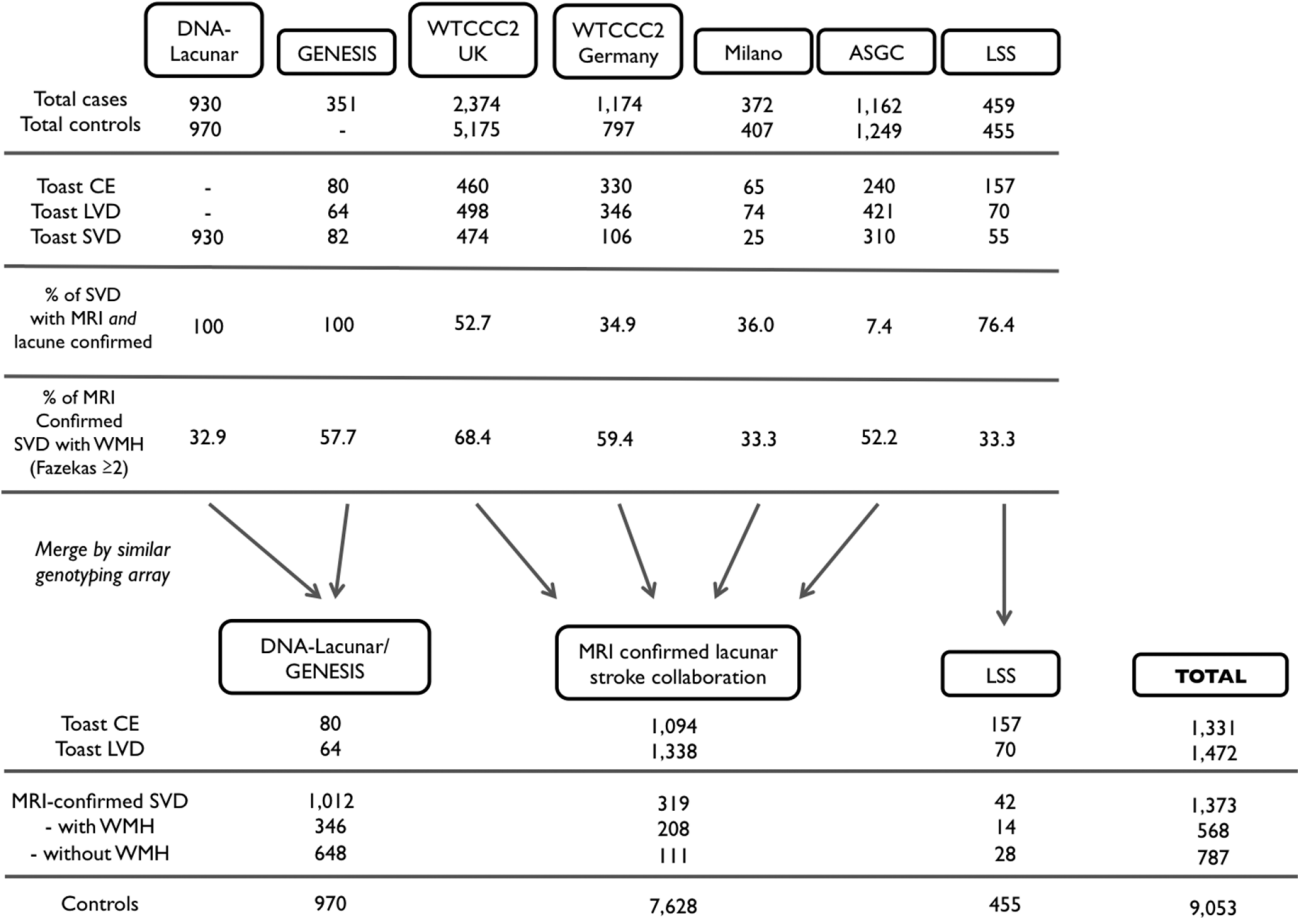
Cohort characteristics. ASGC indicates Australian Stroke Genetics Collaborative; CE, cardioembolic stroke; GENESIS, Genes and Ischaemic Stroke; LSS, Leuven Stroke Study; LVD, large vessel disease; MRI, magnetic resonance imaging; SVD, small vessel disease; WMH, white matter hyperintensities; and WTCCC2, Wellcome Trust Case Control Consortium 2.

The final cohort consisted of 4176 stroke cases, including 1,373 lacunar stroke cases (mean age [SD]=60.0 [11.3] years; 68.0% men), subtyped into 568 with WMH (mean age [SD]=65.1 [10.9] years; 65.0% men) and 787 without WMH (mean age [SD]=56.0 [9.9] years; 70.2% men), 1,331 cardioembolic strokes (mean age [SD]=72.8 [10.6] years; 52.7% men), and 1,472 large vessel strokes (mean age [SD]=66.9 [11.1] years; 67.3% men) and 9,053 controls (mean age [SD]=58.4 [10.7] years [age not available in 2437 of WTCCC2-UK controls]; 52.2% men). Information on WMH volumes was not available in controls. As inclusion in the MRI-informed lacunar stroke analysis depended on the availability of an MRI and confirmation of a lacunar infarct, proportions of lacunar stroke cases varied greatly between studies (Figure 1).

### Genetic Risk Score Analyses

**Figure 2.**
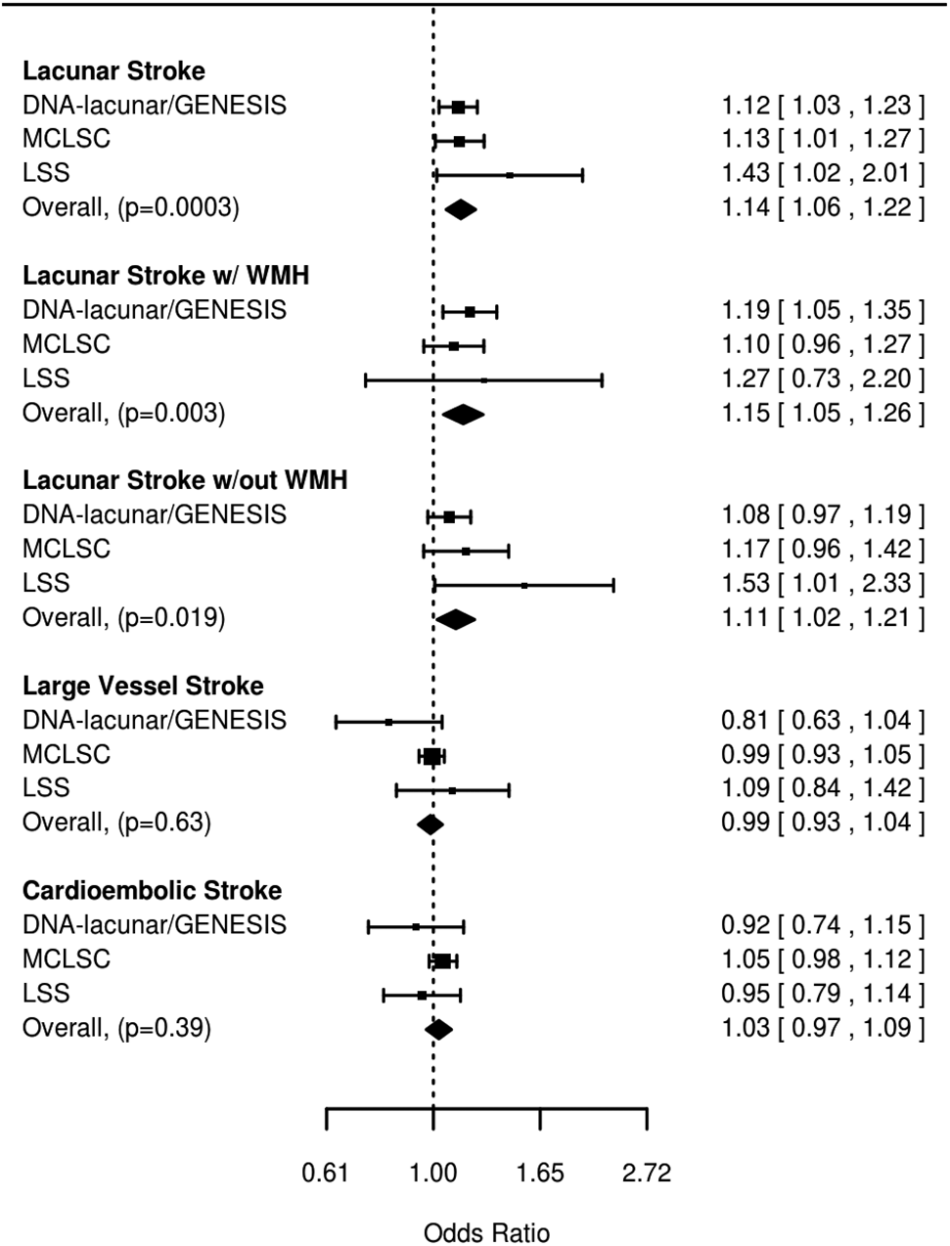
Association of white matter hyperintensities (WMH) Genetic Risk Score with ischemic stroke subtypes. GENESIS indicates Genes and Ischaemic Stroke; LSS, Leuven Stroke Study; and MCLSC, magnetic resonance imaging-confirmed lacunar stroke collaboration (Australian Stroke Genetics Collaborative [ASGC], Wellcome Trust Case Control Consortium 2 [WTCCC2-UK], Wellcome Trust Case Control Consortium 2 [WTCCC2]-Germany and Milano studies).

A genetic risk score comprises 15 SNPs associated with WMH in community populations was significantly associated with lacunar stroke (OR [95% confidence interval [CI]=1.14 [1.06–1.22]; *P*=0.0003; Figure 2). The association was slightly stronger, although not significantly so, in the group with WMH (OR [95% CI]=1.15 [1.05–1.26]; *P*=0.003) and slightly weaker and not reaching Bonferroni-corrected significance in the group without substantial WMH (OR [95% CI]=1.11 [1.02–1.21]; *P*=0.019). Conversely, the risk score was not associated with cardioembolic (OR [95% CI]=1.03 [0.97–1.09]; *P*=0.39) or large vessel stroke (OR [95% CI]=0.99 [0.93–1.04]; *P*=0.63).

### Single SNP Analyses

No SNP reached the a priori significance threshold after Bonferroni correction (Table). Four SNPs (rs7909791[*SH3PXD2A*], OR [95% CI]=1.14 [1.03–1.27]; *P*=0.01; rs72934505[*NBEAL1*], OR [95% CI]=1.24 [1.07–1.45]; *P*=0.004; rs941898[*EVL*], OR [95% CI]=1.12 [1.01–1.25]; *P*=0.04; and rs9515201[*COL4A2*], OR [95% CI]=1.15 [1.03–1.27]; *P*=0.01) reached a nominal significance threshold in the all lacunar stroke analysis.

## Discussion

We used a genetic risk score approach to determine whether genetic variants associated with WMH confer risk of lacunar stroke, and therefore whether WMH and lacunar stroke share pathogenesis. We found strong evidence that genetic variants associated with WMH in community populations also influence risk of lacunar stroke. This provides further evidence to support the long-held view that neuroimaging features of cerebral SVD share pathophysiology. When dividing our lacunar stroke population into those with moderate to severe WMH and those without, we found some evidence for association with both groups, although the association was marginally (and not significantly) stronger in the group with WMH and the association in the group without WMH did not reach Bonferroni-corrected significance. This suggests that variants influencing WMH confer risk of lacunar stroke even for lacunar strokes without substantial WMH. In addition, 2 of the SNPs, rs9515201[*COL4A2*] and rs2984613[*PMF1-BGLAP*], are also associated with intracerebral hemorrhage.^10,22^ This serves to emphasize that shared pathophysiological processes seem to underlie many of the clinical manifestations of cerebral SVD and suggests that a coordinated attempt to identify cerebral SVD associations will likely be fruitful. Four SNPs reached nominal significance for association with lacunar stroke (rs7909791[*SH3PXD2A*], rs72934505[*NBEAL1*], rs941898[*EVL*], and rs9515201[*COL4A2*]). With the exception of *COL4A2*, which has been linked to SVD, none of these loci have formerly been linked to ischemic stroke. As discussed above, similar arterial changes have been described in patients with lacunar stroke or WMH,^5^ including diffuse arteriosclerosis and a more focal microatheroma. Other studies have shown that mechanisms including blood–brain barrier dysfunction,^22–24^ and endothelial dysfunction,^25^ are important in both.^23^ As our results show a shared molecular basis to the 2 traits, they might suggest that these findings are because of the fact that WMH and lacunar stroke are outward manifestations of a shared underlying pathological process, namely, cerebral SVD.

In contrast, a genetic risk score comprising the same 15 SNPs was not associated with large vessel or cardioembolic stroke. Some studies have shown a relationship between subclinical atherosclerosis and WMH,^23,24^ whereas others have found an increased risk of all stroke in individuals with WMH.^25^ Our results, in a well-characterized population, suggest that the relationship between WMH and ischemic stroke is limited to lacunar stroke. This finding might suggest that previously reported associations between nonlacunar strokes and WMH may be because of shared risk factors such as hypertension rather than shared pathogenesis.

This study has several strengths. The sample size was large and all lacunar strokes were confirmed by MRI, reducing the possibility of misclassification which might occur when using CT. In addition, the design of the study, which made use of genetic data, means that the results are less susceptible to the residual confounding and reverse causation that observational studies can suffer from, although other sources of confounding, such as technical artifacts, may arise. Similarly, this study has weaknesses. We were unable to evaluate 3 rare SNPs which were associated with WMH in the previous publication,^9^ which may have affected our results. Some lacunar infarcts were diagnosed as acute lesions on DWI, but others were diagnosed from MRI scans performed after the acute stroke phase as cavities on T1 or fluid-attenuated inversion recovery images. The inclusion of patients defined using these different radiological criteria may introduce a subtle bias, and it is possible that some of these cavities could have resulted from hemorrhage rather than ischemia. Although MRI was performed for all lacunar stroke subjects, it was not performed in all nonlacunar subtypes, which could lead to some degree of misclassification. In addition, controls were historical and MRI was not performed to rule out cerebrovascular disease.

## Conclusions

Genetic factors that affect WMH are also associated with risk of lacunar stroke, but not other stroke subtypes. This sheds new light on processes that are implicated in lacunar stroke and provides further evidence that shared pathophysiological processes underlie different manifestations of SVD.

## Sources of Funding

H. Markus was supported by an National Institure for Health Research (NIHR) Senior Investigator award. H. Markus and Dr Bevan were supported by the NIHR Cambridge University Hospitals Comprehensive Biomedical Research Centre. Collection of the UK Young Lacunar Stroke Resource was primarily supported by the Wellcome Trust (WT072952) with additional support from the Stroke Association (TSA 2010/01). Genotyping and Dr Traylor were supported by a project grant from the Stroke Association (TSA 2013/01). The research was also supported by the NIHR Biomedical Research Centre based at Guy’s and St Thomas’ NHS Foundation Trust and King’s College London. Dr Thijs was supported by a Flemish Fund of Scientific Research (FWO) Clinical Investigator Grant.

## Disclosures

None.

## Supplementary Material
