# Supplementary material for: Genetic Associations With White Matter Hyperintensities Confer Risk of Lacunar Stroke
Source: Stroke. 2016 Apr 25;47(5):1174–9. doi: 10.1161/STROKEAHA.115.011625 (PMC4839546; doi:10.1161/STROKEAHA.115.011625)
Supplement: Supplementary file 1 [file str-47-1174-s001.pdf]

SUPPLEMENTAL MATERIAL

Genotyping and imputation

Cohorts genotyped on the Illumina 610K (Australian cases and controls, Italian cases), 660W (UK cases and controls, German cases and Italian controls) and 1M arrays (German controls) were treated as one batch. Quality control was performed on each dataset separately, as previously described. [1-3] A consensus set of 381,428 SNPs was then identified that was consistent across the four populations and the four populations were merged for these SNPs. We then performed principal components analysis using EIGENSTRAT on an LD-pruned set of SNPs from the combined dataset removing any population outliers, [4] defined as greater than 6 standard deviations from the mean on the first 5 principal components. 37 individuals were removed in total (29 Australian, 8 Italian; 31 cases, 6 controls). The remaining individuals were then imputed to 1000 Genomes phase 3:[5] SHAPEIT v2 was used to phase the haplotypes and IMPUTE v2.2.2 was used to perform the imputation.[6, 7]

The DNA-lacunar dataset was genotyped on the Illumina HumanExomeCore array. This array contains both exome content (~250,000 SNPs) and common tag SNPs (~250,000 SNPs) found on conventional GWAS arrays. Post-imputation, this array provides comparable coverage of common SNPs to larger arrays (MAF>5%, 78.2% $r^2 \geq 0.8$ compared to 86.6% for Illumina OmniExpress).[8] SNPs were excluded with MAF<0.01, genotype missingness>3%, HWE p<1e-6 in controls, strand ambiguity (A/T or C/G) or evidence of differential missingness by case-control status (p<0.05). Individuals were excluded if they had missingness>3%, excess or reduced heterozygosity, showed evidence of relatedness with another individual (pi-hat>0.1875), or failed a "sex-check" in PLINK. EIGENSTRAT was used to remove non-caucasian individuals, and was then repeated to calculate ancestry-informative principal components.[4] The remaining 269,691 autosomal SNPs and 2,603 individuals were then imputed to 1000 Genomes phase 3:[5] SHAPEIT v2 was used to phase the haplotypes and IMPUTE v2.2.2 was used to perform the imputation.[6, 7]

The Leuven dataset (Leuven Stroke Study) was genotyped on the Illumina Omni 5M array. SNPs were excluded with MAF<0.01, genotype missingness>3%, HWE p<1e-6 in controls, strand ambiguity (A/T or C/G) or evidence of differential missingness by case-control status (p<0.05). Individuals were excluded if they had missingness>3%, excess or reduced heterozygosity, showed evidence of relatedness with another individual (pi-hat>0.1875), or failed a "sex-check" in PLINK. EIGENSTRAT was used to remove non-caucasian individuals. The remaining individuals were then imputed to 1000 Genomes phase 3:[5] SHAPEIT v2 was used to phase the haplotypes and IMPUTE v2.2.2 was used to perform the imputation.[6, 7]

References

1. Holliday EG, Maguire JM, Evans TJ, Koblar SA, Jannes J, Sturm JW, et al. Common variants at 6p21.1 are associated with large artery atherosclerotic stroke. *Nat. Genet.* 2012;44:1147-1151
2. Bellenguez C, Bevan S, Gschwendtner A, Spencer CC, Burgess AI, Pirinen M, et al. Genome-wide association study identifies a variant in hdac9 associated with large vessel ischemic stroke. *Nat. Genet.* 2012;44:328-333
3. Traylor M, Farrall M, Holliday EG, Sudlow C, Hopewell JC, Cheng YC, et al. Genetic risk factors for ischaemic stroke and its subtypes (the metastroke collaboration): A meta-analysis of genome-wide association studies. *Lancet Neurol*. 2012;11:951-962
4. Price AL, Patterson NJ, Plenge RM, Weinblatt ME, Shadick NA, Reich D. Principal components analysis corrects for stratification in genome-wide association studies. *Nat. Genet.* 2006;38:904-909
5. Abecasis GR, Auton A, Brooks LD, DePristo MA, Durbin RM, Handsaker RE, et al. An integrated map of genetic variation from 1,092 human genomes. *Nature*. 2012;491:56-65
6. O'Connell J, Gurdasani D, Delaneau O, Pirastu N, Ulivi S, Cocca M, et al. A general approach for haplotype phasing across the full spectrum of relatedness. *PLoS Genet*. 2014;10:e1004234
7. Howie BN, Donnelly P, Marchini J. A flexible and accurate genotype imputation method for the next generation of genome-wide association studies. *PLoS Genet*. 2009;5:e1000529
8. Nelson SC, Doheny KF, Pugh EW, Romm JM, Ling H, Laurie CA, et al. Imputation-based genomic coverage assessments of current human genotyping arrays. *G3 (Bethesda, Md.)*. 2013;3:1795-1807

Appendix

UK Young Lacunar Stroke DNA Study collaborators

Study managers: Josie Monaghan; Alan Zanich, Samantha Febrey, Eithne Smith, Jenny Lennon, St George's University of London

Database cleaning: Loes Rutten-Jacobs, University of Cambridge

Participating centres (number of enrolled patients per centre; local investigators):

Aberdeen Royal Infirmary, Aberdeen (12; Mary Macleod). Addenbrooke's Hospital, Cambridge (54; Jean-Claude Baron, Elizabeth Warburton, Diana J Day, Julie White). Airedale General Hospital, Steeton (4; Samantha Mawer). Barnsley Hospital, Barnsley (3; Mohammad Albazzaz, Pravin Torane, Keith Elliott, Kay Hawley). Bart's and the London, London (2; Patrick Gompertz). Basingstoke and North Hampshire Hospital, Basingstoke (13; Elio Giallombardo, Deborah Dellafera). Blackpool Victoria Hospital, Blackpool (11; Mark O'Donnell). Bradford Royal Infirmary, Bradford (1; Chris Patterson). Bristol Royal Infirmary, Bristol (8; Sarah Caine). Charing Cross Hospital, London (12; Pankaj Sharma). Cheltenham General and Gloucester Royal Hospitals, Cheltenham and Gloucester (10; Dipankar Dutta). Chesterfield Royal Hospital, Chesterfield (4; Sunil Punnoose, Mahmud Sajid). Countess of Chester Hospital, Chester (22; Kausik Chatterjee). Derriford Hospital, Plymouth (4; Azlisham Mohd Nor). Dorset County Hospital NHS Foundation Trust, Dorchester (6; Rob Williams). East Kent Hospitals University NHS Foundation Trust, Kent (22; Hardeep Baht, Guna Gunathilagan). Eastbourne District General Hospital, Eastbourne (4; Conrad Athulathmudali). Frenchay Hospital, Bristol (1; Neil Baldwin). Frimley Park Hospital NHS Foundation Trust, Frimley (6; Brian Clarke). Guy's and St Thomas' Hospital, London (14; Tony Rudd). Institute of Neurology, London (25; Martin Brown). James Paget University Hospital, Great Yarmouth (1; Peter Harrison). King's College Hospital, London (16; Lalit Kalra). Leeds Teaching Hospitals NHS Trust, London (125; Ahamad Hassan). Leicester General Hospital and Royal Infirmary, Leicester (9; Tom Robinson, Amit Mistri). Luton and Dunstable NHSFT University Hospital, Luton (16; Lakshmanan Sekaran, Sakthivel Sethuraman, Frances Justin). Maidstone andTunbridge Wells NHS Trust (3; Peter Maskell). Mayday University Hospital, Croydon (14; Enas Lawrence). Medway Maritime Hospital, Gillingham (5; Sam Sanmuganathan). Milton Keynes Hospital, Milton Keynes (1; Yaw Duodu). Musgrove Park Hospital, Taunton (9; Malik Hussain). Newcastle Hospitals NHS Foundation Trust, Newcastle upon Tyne (12; Gary Ford). Ninewells Hospital, Dundee (5; Ronald MacWalter). North Devon District Hospital, Barnstaple (8; Mervyn Dent). Nottingham University Hospitals, Nottingham (17; Philip Bath, Fiona Hammonds). Perth Royal Infirmary, Perth (2; Stuart Johnston). Peterborough City Hospital, Peterborough (1; Peter Owusu-Agyei). Queen Elizabeth Hospital, Gateshead (5; Tim Cassidy, Maria Bokhari). Radcliffe Infirmary, Oxford (5; Peter Rothwell). Rochdale Infirmary, Rochdale (4; Robert Namushi). Rotherham General Hospital, Rotherham (1; James Okwera). Royal Cornwall Hospitals NHS Trust, Truro (11; Frances Harrington, Gillian Courtauld). Royal Devon and Exeter Hospital, Exeter (22; Martin James). Royal Hallamshire Hospital, Sheffield (1; Graham Venables). Royal Liverpool University Hospital and Broadgreen Hospital, Liverpool (9; Aravind Manoj). Royal Preston Hospital, Preston (18; Shuja Punekar). Royal Surrey County Hospital, Guildford (23; Adrian Blight, Kath Pasco). Royal Sussex County Hospital, Brighton (14; Chakravarthi Rajkumar, Joanna Breeds). Royal United Hospital, Bath (6; Louise Shaw, Barbara Madigan). Salford Royal Hospital, Salford (16; Jane Molloy). Southampton General Hospital, Southampton (1; Giles Durward). Southend Hospital, Westcliff-on-Sea (26; Paul Guyler). Southern General Hospital, Glasgow (34; Keith Muir, Wilma Smith). St George's Hospital, London (108; Hugh Markus). St Helier Hospital, Carshalton (10; Val Jones). Stepping Hill Hospital, Stockport (4; Shivakumar Krishnamoorthy). Sunderland Royal Hospital, Sunderland (1; Nikhil Majumdar). The Royal Bournemouth Hospital, Bournemouth (15; Damian Jenkinson). The Walton Centre, Liverpool (15; Richard White). Torbay Hospital, Torquay (19; Debs Kelly). University Hospital Aintree, Liverpool (19; Ramesh Durairaj). University Hospital of North Staffordshire, Stoke-on-trent (16; David Wilcock). Wansbeck General Hospital and North Tyneside Hospital, Ashington and North Shields (6; Christopher Price). West Cumberland

Hospital, Whitehaven (6; Olu Orugun, Rachel Glover). West Hertfordshire Hospital, Watford (20; David Collas). Western General Hospital, Edinburgh (12; Cathie Sudlow). Western Infirmary, Glasgow (33; Kennedy R. Lees, Jesse Dawson). Wycombe Hospital and Stoke Mandeville, High Wycombe (20; Dennis Briley and Matthew Burn). Yeovil District Hospital, Yeovil (46; Khalid Rashed). York Teaching Hospital, York (1; John Coyle).
